# A research agenda for burn infection prevention: identifying knowledge gaps and prioritizing future directions

**DOI:** 10.1017/ash.2025.10213

**Published:** 2025-10-30

**Authors:** Madhuri M. Sopirala, David Weber, Geeta Sood, Mohamed Yassin, Tina L. Palmieri, Supriya Narasimhan, Clifford Sheckter, Julie Caffrey, Samuel Mandell, Larissa Pisney, Sheetal Kandiah, Jyoti Somani, Natalie Mackow, Karen Brust, Sara Karaba, Michelle Doll, Donald Chen, Lilian Abbo, Werner Bischoff

**Affiliations:** 1 Division of Infectious Diseases and Geographic Medicine, Department of Internal Medicine, University of Texas Southwestern Medical Center, Dallas, TX, USA; 2 Parkland Health, Dallas, TX, USA; 3 Division of Infectious Diseases, Department of Medicine, University of North Carolina School of Medicine, Chapel Hill, NC, USA; 4 Department of Medicine, Johns Hopkins School of Medicine, Baltimore, MD, USA; 5 University of Pittsburgh School of Medicine, University of Pittsburgh, Pittsburgh, PA, USA; 6 Burn Division, Department of Surgery, University of California, Davis and Shriners Children’s Northern California, Sacramento, CA, USA; 7 Department of Medicine, Santa Clara Valley Medical Center, San Jose, CA, USA; 8 Department of Medicine, Division of ID, Stanford University, Stanford, CA, USA; 9 Department of Surgery, Santa Clara Valley Medical Center, San Jose, CA, USA; 10 Department of Surgery, Stanford University, Stanford, CA, USA; 11 Bayview Burn Center, Department of Plastic and Reconstructive Surgery, Johns Hopkins University, Baltimore, MD, USA; 12 Department of Surgery, University of Texas Southwestern Medical Center, Dallas, TX, USA; 13 Burn Program, Parkland Health, Dallas, TX, USA; 14 Division of Infectious Diseases, University of Colorado School of Medicine, Aurora, CO, USA; 15 Division of Infectious Diseases, Emory University School of Medicine, Atlanta, GA, USA; 16 Grady Health System, Atlanta, GA, USA; 17 Infection Prevention and Control Program, Jackson Health System, Miami, FL, USA; 18 Division of Infectious Diseases, Department of Medicine, University of Miami Miller School of Medicine, Miami, FL, USA; 19 University of Iowa Health Care, Iowa City, IA, USA; 20 VCU School of Medicine, Richmond, VA, USA; 21 VCU Health System, Richmond, VA, USA; 22 Division of Infectious Diseases; Infection Prevention, Westchester Medical Center, Valhalla, NY, USA; 23 Section of Infectious Diseases, Department of Internal Medicine, Wake Forest University School of Medicine, Winston-Salem, NC, USA

## Abstract

**Objective:**

Burn injuries result in loss of skin barrier and altered immune responses that in turn make patients especially vulnerable to healthcare-associated infections. Despite prolonged exposures of these patients to hospital environments, burn-specific infection prevention strategies are understudied. We present a research agenda identifying key research gaps and organizing them into priority areas to guide future investigations in this high-risk population.

**Design:**

Members of the Society for Healthcare Epidemiology of America (SHEA) Burn Infection Prevention and Control Special Interest Group and the American Burn Association (ABA) collaborated to develop this research agenda, combining expertise in infection prevention, antimicrobial stewardship, and burn care.

**Results:**

We identified five priority areas: (1) improving surveillance and epidemiologic data on burn infections; (2) better understanding of microbiology, including biofilms and the microbiome; (3) evaluating wound healing strategies; (4) refining infection prevention and control practices unique to burn units; and (5) building burn patient specific risk assessment and predictive models. The agenda highlights the need for standardized definitions and shared data platforms. It calls for evaluation of practical strategies for infection prevention, stewardship, and environmental control.

**Conclusions:**

This research agenda intends to help guide future studies aimed at furthering knowledge and improving outcomes in burn care.

Infection remains the single greatest cause of mortality for admitted burn patients.^1^ Preventing infections is therefore critical to improving patient outcomes and reducing healthcare costs.^1^ Burn injuries are the fourth most common form of trauma worldwide, affecting approximately 11 million people each year.^2^ There are varying reports of the burden related to burn injury in the United States. According to ABA, approximately half a million people sustain burn injuries requiring medical intervention. Around 40,000 of these people are admitted, with three-quarters of them needing specialized treatment at a certified burn center.^1,3^ According to the 2020 United States National Inpatient Sample (NIS data), there were approximately 89,555 inpatient stays with a burn diagnosis and 29,165 inpatient stays with a burn-related diagnosis-related group (DRG) code, resulting in an estimated total of 118,720 inpatient stays for that year.^3,4^ The 2020 US Nationwide Emergency Department Sample (NEDS) data estimated that 438,185 patients were evaluated in the emergency department (ED) with burn-related diagnosis codes.^4^ Although overall mortality remains relatively low, outcomes worsen substantially in cases involving complications such as inhalation injury or extended ICU stay.^4^ Infectious complications range from 20% to 60%.^3,5^ In a scoping review examining the impact of infection on length of stay in adult burns, 81% (13/16 studies) reported an increase greater than 1.5-fold, and 50% (8/16 studies) reported an increase greater than 2-fold for infection.^6^ Length of stay for burn patients with surgical wound infection is three times higher with a mean of 91.4 days compared to 31.7 days in an uninfected patient.^7^

Despite the burden posed by these infections, there are substantial gaps in our understanding of infection prevention in burn patients. A group of healthcare epidemiologists from the Society of Healthcare Epidemiology of America (SHEA)’s Burn Infection Prevention and Control Special Interest Group and burn surgeons from ABA have collaborated to propose the following list of priority areas that are critical in providing timely and adequate protection for burn patients to be considered for further research. The proposed objectives under each priority area are intended as a flexible resource for researchers, whether working independently or through collaborative efforts across institutions. Carrying out these objectives may require IRB approval and research funding, depending on study design and scope.

## Priority area #1

### Epidemiology of burn infections

Data from single center studies and the Burn Care Quality Platform (BCQP) show worse outcomes in burn patients with infections.^8–16^ Although these data show a consistent adverse association, there remains significant variability in reported infection rates due to inconsistencies in case definitions and surveillance approaches.^6^ Infections in burn patients can be challenging to diagnose due to patients’ altered physiological response, which may include elevated baseline body temperatures and inflammation, masking or mimicking signs of infection.^17^ Even though research studies have examined the incidence of certain pathogens such as methicillin-resistant *Staphylococcus aureus* (MRSA) and *Pseudomonas aeruginosa*, data on emerging pathogens and emerging patterns of resistance remain limited.^18,19^

Although the BCQP captures valuable burn-related outcomes, it is not primarily focused on infection surveillance. To enable consistent, comparable, and generalizable data across institutions, future research should focus on establishing consensus-based definitions jointly developed by the ABA, Centers for Disease Control and Prevention (CDC), and infection prevention experts. This consensus would lay the foundation for a dedicated, standardized infection surveillance database that complements existing registries like BCQP and supports benchmarking, quality improvement, and research across burn units nationwide.

A major research gap exists in the standardized assessment of prevalence, incidence, and risk factors for burn infections including those caused by drug-resistant pathogens and in understanding their impact on healthcare outcomes.

Objective 1: Develop harmonized infection definitions that are standardized between CDC/National Health Safety Network (CDC/NHSN) and ABA to enable consistent surveillance of infections in burn patients across various settings such as intensive care, step-down units, and rehabilitation settings. This surveillance should include surgical wound infections, bloodstream infections, respiratory infections, and urinary tract infections.

Objective 2: design and implement a dedicated, standardized infection surveillance database for burn units, informed by consensus definitions. This database should include data on patient demographics, comorbidities, burn type, and severity, long-term outcomes such as quality of life, functional outcomes, and mortality, along with unit-level variables such as unit design, environmental controls, unit policies and procedures and staffing.

Objective 3: Establish real-time surveillance systems for infections and antimicrobial resistance trends in burn units to inform infection prevention strategies and guide empiric therapy.

Collaborative research between burn surgery, infectious diseases, and public health agencies will be essential to break down existing silos and develop a unified framework for infection surveillance and benchmarking in burn care.

## Priority area #2: microbiology of burn infections

Burn infections and wound healing are likely influenced not just by single microbial organism, but communities of microorganisms and their interactions.

In vitro and in vivo models have been used to study the composition of biofilms, and there have been a few studies exploring biofilm forming bacteria in burn patients.^20–22^ In an in vitro model, bacterial pathogens *Staphylococcus aureus* and *Pseudomonas aeruginosa* survived as coexisting organisms while the fungal pathogen was largely eliminated from the wound site.^23^ Wound biofilm infection led to increased inflammation and burn depth progression which in turn resulted in an invasive infection.^23^ Literature on the relationship of biofilms and the development of invasive infections in burn patients is lacking. Very little is known about the microbiota after burn injury. This is especially true of the airway and skin microbiota in burn patients.^24^ Some studies have suggested that impaired wound healing is associated with dysbiosis of the skin microbiota, but the effects of burn injury on the skin microbiome are not fully understood.^25–28^ The relationship between microbiome composition and wound healing needs to be studied further.^24^

### Future work should focus on two areas for research under this priority area—microbiome of the burn wound and biofilms

Microbiome: A research gap exists in the understanding of the normal microbiome of the burn wound and the relationship of changes in microbiome with infections and wound healing in burn patients.

Objective 1: Study the microbiome changes caused by burn injuries, excisional surgeries, and topical treatments and the resultant effect on infections.

Objective 2: Investigate the relationship between microbiome composition of the burn wound, metabolomic profiles, and the development of invasive infections, including those associated with indwelling devices.

Biofilms: A research gap exists in the interplay of biofilms and development of infections and wound healing in burn patients.

Objective 1: Study the time line of emergence of most common pathogens such as *Staphylococcus aureus*, *Pseudomonas aeruginosa,* and *Candida* spp. in wound biofilms in burn patients.

Objective 2: Investigate the frequency and time line of emergence of various pathogens including multidrug-resistant organisms such as carbapenem-resistant *Enterobacterales* (CRE) and *P. aeruginosa*, *Candida auris*, molds such as *Aspergillus*, *Rhizopus,* and *Fusarium* spp. within biofilms in relation to antibiotic use.

Objective 3: Investigate the frequency and time line of emergence of the above pathogens within biofilms in relation to different types of wound care practices such as frequency, use of topical antimicrobials and various antiseptics, hydrotherapy practices such as water disinfection and use of point-of-care filters and unit design.

Objective 4: Investigate the relationship between biofilm formation and the development of invasive infections, including those associated with indwelling devices.

Objective 5: Investigate the resistance of biofilms to conventional treatments and explore the role of unconventional treatments such as phages and nanoparticles in treatment and prevention of infections in burn patients.

## Priority area #3

### Wound healing

Healing of the protective skin barrier in burned patients is critical to prevent infections. Many surgical and medical treatments have been used to aid the healing process. More robust randomized control trials are needed to compare various surgical and medical treatments used in burn units.

Objective 1: Test wound healing treatments and devices in burn patients such as microbiocidal creams, cellular and/or tissue-based products for wounds, and negative pressure dressings. Randomized clinical trials that would enable comparison of different treatment methods are needed.

Objective 2: Develop guidance on best surgical approaches to promote wound healing, including timing and staging.

## Priority area #4

### Infection prevention and control strategies

A large research gap exists with regards to infection prevention in burn units. Knowledge driven from other patient populations are generally applied to the burn units. However, the burn population is different, with very different risk factors including a heavy wound and environmental microbial bioburden in the setting of significant immunosuppression and loss of protective biologic barriers. Infection prevention and control strategies must account for these specific challenges including basic hand hygiene and isolation practices, environmental control measures, antimicrobial and diagnostic stewardship, and wound management.

Staff practices of hand hygiene and isolation precautions are paramount as the environment is considered to play a major role in infections in these patients.^29–31^ These are supported by environmental control measures such as surface and equipment cleaning and disinfection, but also extend to unit/room design, air quality, and water quality.^32,33^ However, literature is limited on air and water quality specific to burn units, mostly addressing outbreak situations. Due in part to heavy antimicrobial use in these patients, multidrug resistance is rampant.^34^

Prevention practices for device-related infections in burn units vary significantly, reflecting a lack of standardized guidelines tailored to this unique patient population. Strategies effective in other patient groups are often applied to burn patients, but their non-intact skin and limited anatomical access require adapted approaches. Variability in practices, such as intravenous and urinary catheter exchange protocols and bathing procedures, highlights the need for further research.

Studies need to be performed examining the effect of collaboration between burn surgeons and infectious diseases physicians to proactively pursue effective and safe treatment option as part of antimicrobial stewardship efforts. Diagnostic stewardship has the potential to further optimize treatment plans. This includes but is not limited to pathogen screening (eg, MRSA, CRE) and preventive treatment efforts (eg, decolonization) using appropriate and timely testing such as rapid diagnostic technologies to identify infectious complications.^35^ Wound management techniques play an important role in preventing infections but the individual effect of those techniques on infections has not been well defined.^36,37^

Objective 1: Assess the effectiveness of infection prevention protocols including hand hygiene protocols, isolation precautions, use of personal protective equipment (PPE), respiratory hygiene, and other infection prevention measures in this specific population

Objective 2: Assess the effectiveness of standard and novel environmental cleaning, high level disinfection and sterilization protocols used for reusable devices in burn patients.

Objective 3: Evaluate the effectiveness of environmental control measures designed to reduce the environmental burden in burn units through unit design, and air purification, water filtration, and surface decontamination systems.

Objective 4: Investigate the variability in device-related infection prevention practices among burn units and identify evidence-based strategies to inform the development of standardized guidelines.

Objective 5: Evaluate the role of diagnostic and antibiotic stewardship programs in optimizing antimicrobial use among burn patients and to assess their impact on clinical outcomes, including the incidence of antimicrobial-resistant colonization and infections.

Objective 6: Study the appropriate use of laboratory testing to guide patient management, including treatment, to optimize clinical outcomes and limit the spread of antimicrobial resistance.

Objective 7: Study infection outcomes in burn patients managed through a collaborative management model of Burn Surgery and Infectious Diseases physicians.

Objective 8: Research existing practices for wound management, including different types of dressings, debridement techniques, topical antimicrobials, and surgical interventions including skin grafts and reconstructive surgeries.

## Priority area #5

### Risk assessment models

Although risk assessment and risk prediction models for infections exist in general, literature is lacking for burn infections.^38,39^

Understanding specific risk factors for infection in this specialized patient population will allow for more individualized patient prevention strategies. Additionally, current risk adjustment models to compare performance will need to include risk factors that are specifically applicable to this patient population such as total body surface area burn and the presence of inhalational injury which are not currently included in healthcare-associated infection models.

Objective 1: Develop and implement risk assessment frameworks to identify potential infection control vulnerabilities in burn care, utilizing tools such as failure modes and effects analyses (FMEA) for process evaluation, and risk matrices, decision trees, and bowtie models for patient-level risk assessment.

Objective 2: Develop and validate predictive models to estimate the likelihood of burn infections, incorporating clinical variables such as burn size, depth, comorbidities, and time to treatment.

### Other promising areas for research

In addition to the aforementioned research topics, several innovative areas show promise for research. Specifically wound healing technologies, health service delivery and access to care, and appropriate use of diagnostic tests are all areas for further research. These include studying the effect of novel technologies such as bioactive dressings, skin substitutes, and growth factors on wound healing and incidence of infections.^36^

Another emerging area for research is the role of telemedicine for early detection of wound infections, treatment initiation and remote monitoring of patient recovery. This may help deliver timely care to burn patients in remote, underserved areas. Progress in the development and evaluation of rapid diagnostic tools such as PCR-based diagnostics or point-of-care microbiology tests for burn patients can guide early and targeted treatments improving patient outcomes.^36,40^

In addition, postacute care and outpatient management and rehabilitation is a promising area of study for burn patients.^41^ Stress and trauma experienced by burn patients, their caregivers and healthcare workers might affect infection risk and recovery. Psychological interventions to improve infection prevention outcomes might prove to be effective. Lastly, researching cost effective infection prevention strategies in low- and middle-income countries and resource-limited settings may be an opportunity to improve burn wound care on a global scale.
